# A case report: renal cystic tumoural calcinosis with ossification and bone marrow formation

**DOI:** 10.1186/s12894-020-00675-6

**Published:** 2020-07-20

**Authors:** Mancheng Xia, Chun Liu, Haosen Yang, Keqiang Yin, Yusheng Wang, Xunan Tong, Siyu Zhang, Weibing Shuang

**Affiliations:** 1 First Clinical Medical College, Shanxi Medical University, No. 85, JieFang South Road, Yingze District, Taiyuan, 030001 Shanxi China; 2 Department of Urology, The First Hospital of Shanxi Medical University, No. 85, JieFang South Road, Yingze District, Taiyuan, 030001 Shanxi China; 3 Kidney Transplantation Centre, Shanxi Second People’s Hospital, Taiyuan, Shanxi China; 4 Department of Pathology, Shanxi People’s Hospital, Taiyuan, Shanxi China

**Keywords:** Cystic tumoural calcinosis, Ossification, Bone marrow formation, Kidney, Lesion resection

## Abstract

**Background:**

Tumoural calcinosis (TC) is a rare disorder characterized by nonneoplastic amorphous calcium deposition that tends to occur in soft tissues around the large joint. Here, we report a case of cystic TC with ossification and bone marrow formation in the kidney.

**Case presentation:**

We report a 63-year-old woman who presented with a complaint of intermittent right lumbar pain for 2 months. Computed tomography (CT) revealed a large cystic lesion on the lateral side of the right kidney, with a circular calcified wall around the lesion, which compressed, deformed and displaced the right kidney. To relieve the symptoms of right lumbar pain, the patient underwent surgical resection of this cystic lesion without partial removal of the renal parenchyma. The pathological results further confirmed the diagnosis of cystic TC with ossification and bone marrow formation in the right kidney. No recurrence was detected 1 year after surgery.

**Conclusions:**

The main differential diagnoses of TC in the kidney are kidney stone, renal tuberculosis, renal cyst with a calcified wall, and tumour. Patients are treated mainly by complete surgical resection of the lesion.

## Background

Tumoural calcinosis (TC) is a rare benign disease that was first named by Inclan and Leon in 1943 [1]. TC often occurs in the soft tissues around large joints in the hip, shoulder, elbow, and knee, as well as occasionally around small joints in the hand, feet, head, and neck [2–4]; no cases have been reported in the kidney. The disease should be differentiated from primary hyperparathyroidism, hypervitaminosis D, gout, vascular calcification in chronic kidney disease, and dystrophic calcification associated with connective tissue disorders [5]. If TC occurs in the kidney, it needs to be differentiated from kidney stones, renal tuberculosis, renal cysts with calcified walls, and tumours and is treated mainly by complete surgical resection of the lesion. To improve the recognition of renal TC, we report herein a case of cystic TC with ossification and bone marrow formation in the kidney.

## Case presentation

**Fig. 1:**
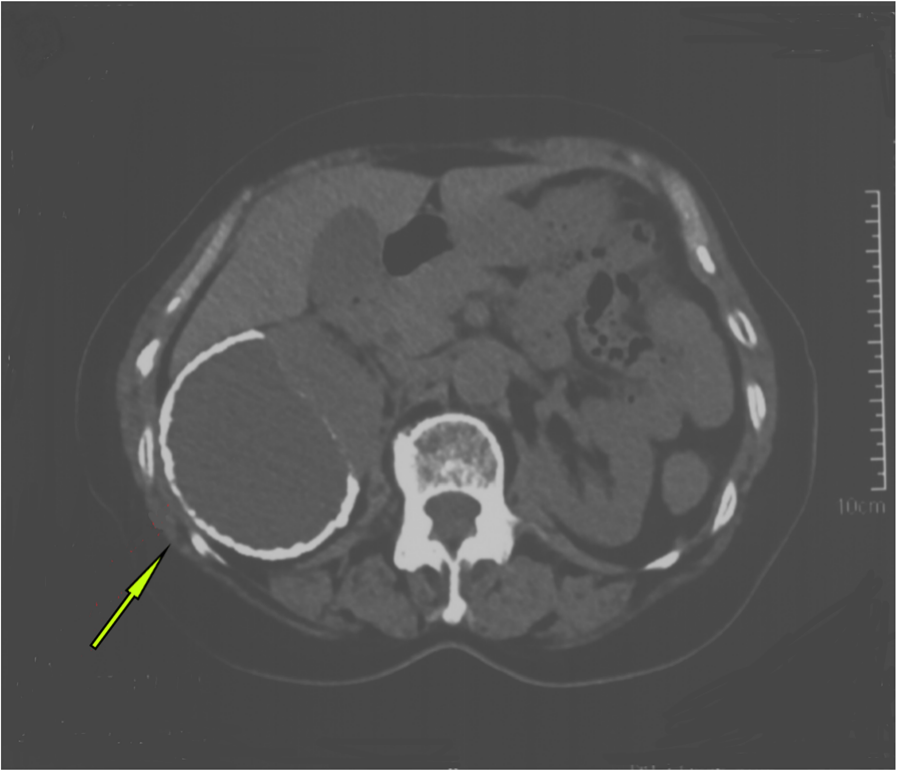
CT scan showing the annular calcification wall around the right kidney, with a size of 11.5 cm*6.9 cm*5.1 cm

**Fig. 2:**
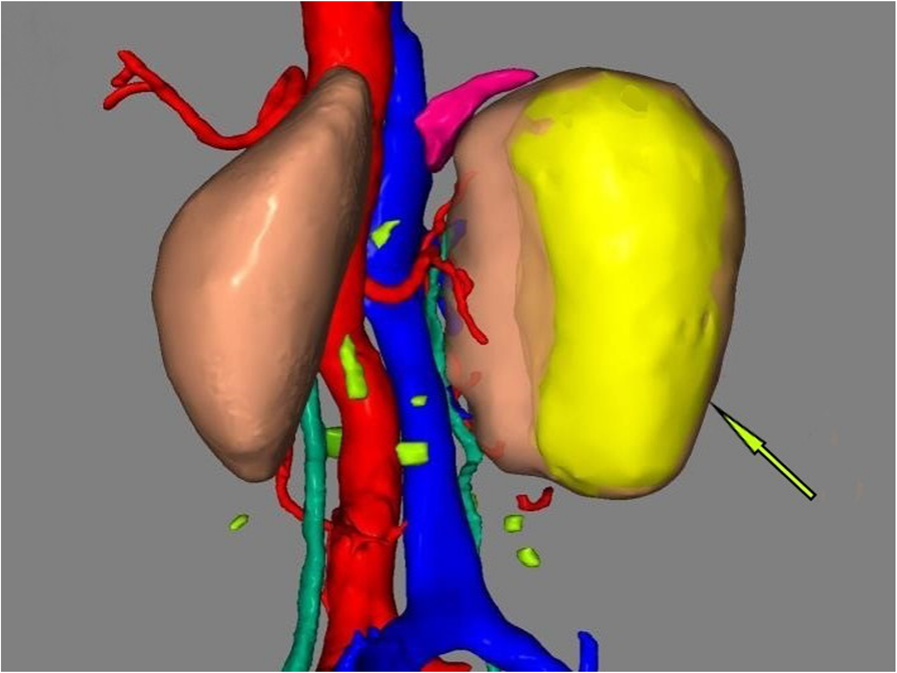
Spiral CT 3D image showing the annular calcification wall around the right kidney, with a size of 11.5 cm * 6.9 cm * 5.1 cm

**Fig. 3:**
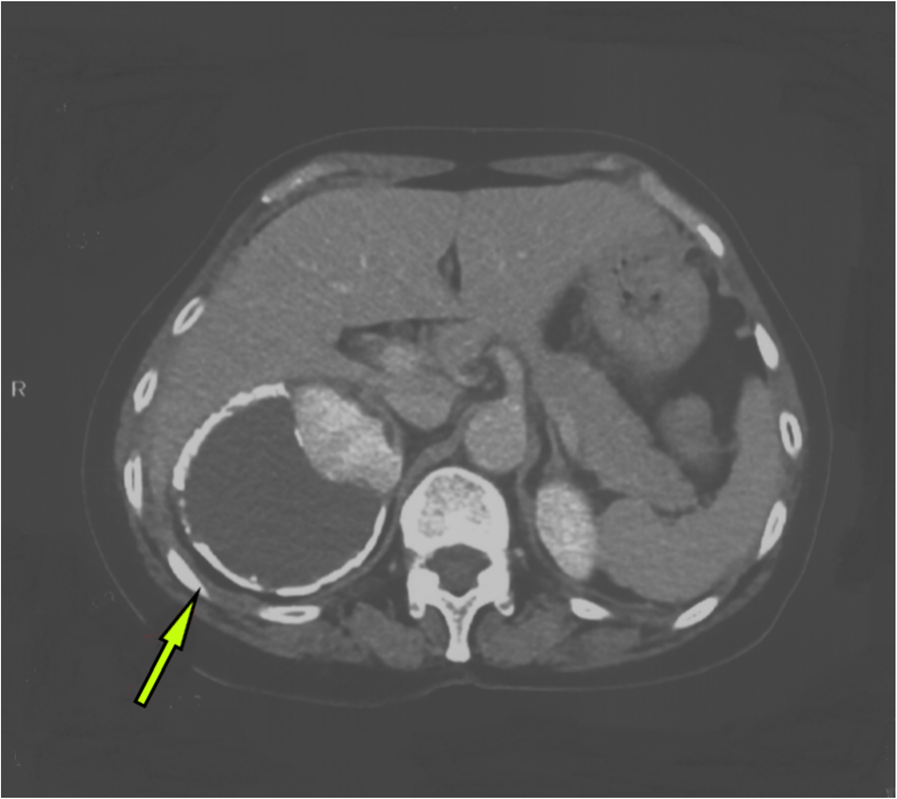
Enhanced CT scan showing a circular low-density shadow under the annular calcification wall

**Fig. 4:**
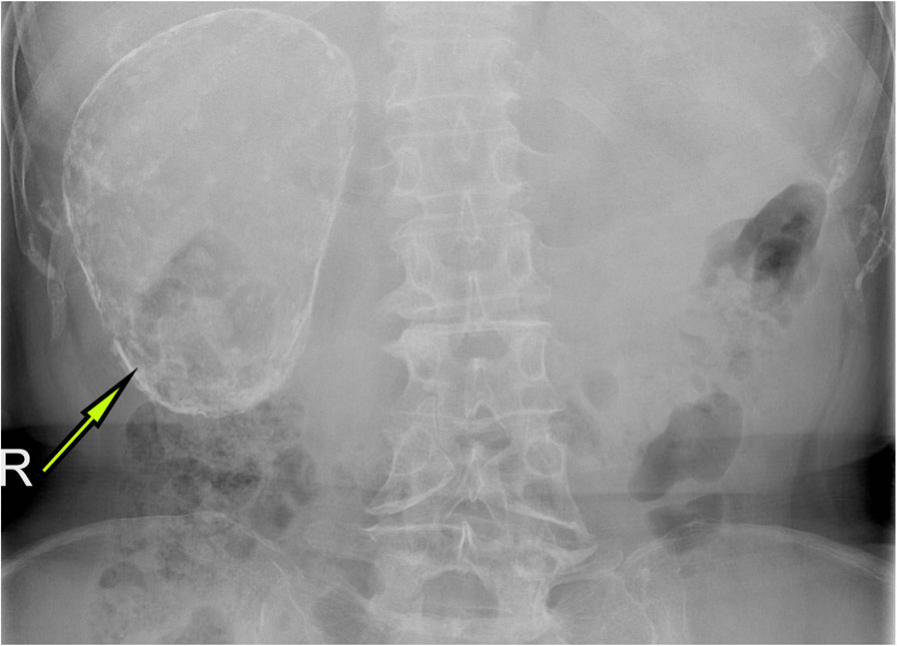
Plain X-ray image of KUB showing the high-density shadow of the right kidney, approximately 10.2 cm * 7.5 cm in size

**Fig. 5:**
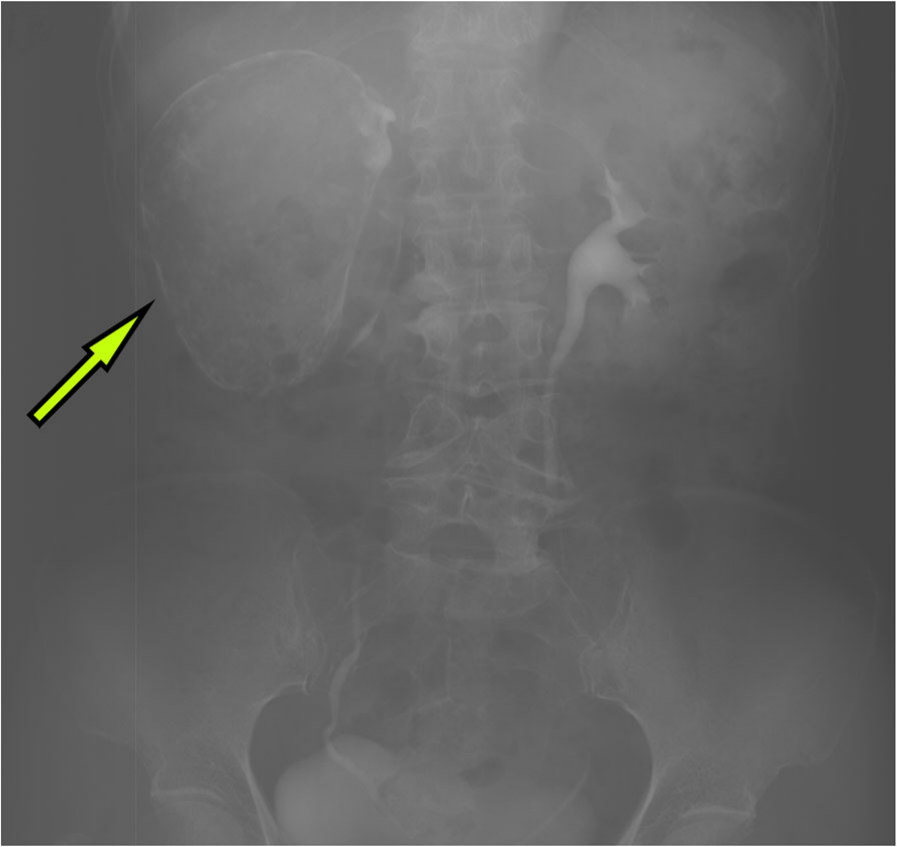
IVU showing only a few minor calyces in the right kidney

A 63-year-old woman was admitted to the hospital with intermittent right lumbar pain, without other symptoms such as fever, weakness, weight loss, and other joint-related symptoms (swelling, joint stiffness, etc.). The patient had a history of right lumbar trauma 2 years prior and hypertension and multiple lacunar infarcts for 6 months. The patient was treated with rosuvastatin calcium (5 mg once daily), compound Danshen tablets (0.96 g once daily), and benazepril hydrochloride (2.5 mg once daily). The patient denied a history of tuberculosis and a related family or consanguinity history of tumoural calcification. Clinical examination revealed no swelling, tenderness and percussive pain in the right lumbar region and no nodules or masses on each joint. Serum phosphate, total calcium, ionized calcium, urea, creatinine, alkaline phosphatase, arterial blood gas analysis, albumin, and uric acid levels were within the normal range (Table 1), and the tuberculin or purified protein derivative (PPD) test result was negative. Computed tomography (CT) scanning and spiral CT 3-D imaging revealed a large cystic lesion on the lateral side of the right kidney, with the circular calcified wall around the lesion, which compressed, deformed and displaced the right kidney. In the annular calcified wall, a low-density circular mass with a size of 11.5 × 6.9 × 5.1 cm and a CT value of 15 HU were identified (Figs. 1 and 2). The density of the circular lesion did not increase in the enhanced CT scan (Fig. 3). Plain X-ray film of the kidneys, ureters and bladder (KUB) showed a high-density shadow in the right waist area measuring 10.2 × 7.5 cm (Fig. 4). In addition, intravenous urography (IVU) revealed that the size of the right kidney decreased, a large cystic lesion was found on the lateral side of the right kidney, and only a few minor calyces were found (Fig. 5).

**Table 1:** Related laboratory tests of this patient

Laboratory tests	Values	Unit	Reference ranges
Serum phosphate	1.14	mmol/L	0.85–1.51
Total calcium	2.41	mmol/L	2.11–2.52
Ionized calcium	1.16	mmol/L	1.15–1.29
Urea	2.94	mmol/L	2.8–7.6
Creatinine	57.4	umol/L	49–90
Alkaline phosphatase	84	U/L	50–135
Albumin	45.7	g/L	40–55
Uric acid levels	206	umol/L	150–350
Arterial blood gas analysis			
PaO_2_	99.9	mmHg	80–100
SaO_2_	97.5	%	95–99
PaCO_2_	35.1	mmHg	35–45
PH	7.44		7.35–7.45
ABE	−0.8	mmol/L	−3 - + 3
SBE	−0.9	mmol/L	−3 - + 3
SBC	23.8	mmol/L	22–27
HCO_3_^−^	23.2	mmol/L	21–28
Anion gap	10.1	mmol/L	8–16

*ABE* Actual base excess, *SBE* Standard base excess, *SBC* Standard bicarbonate radical

**Fig. 6:**
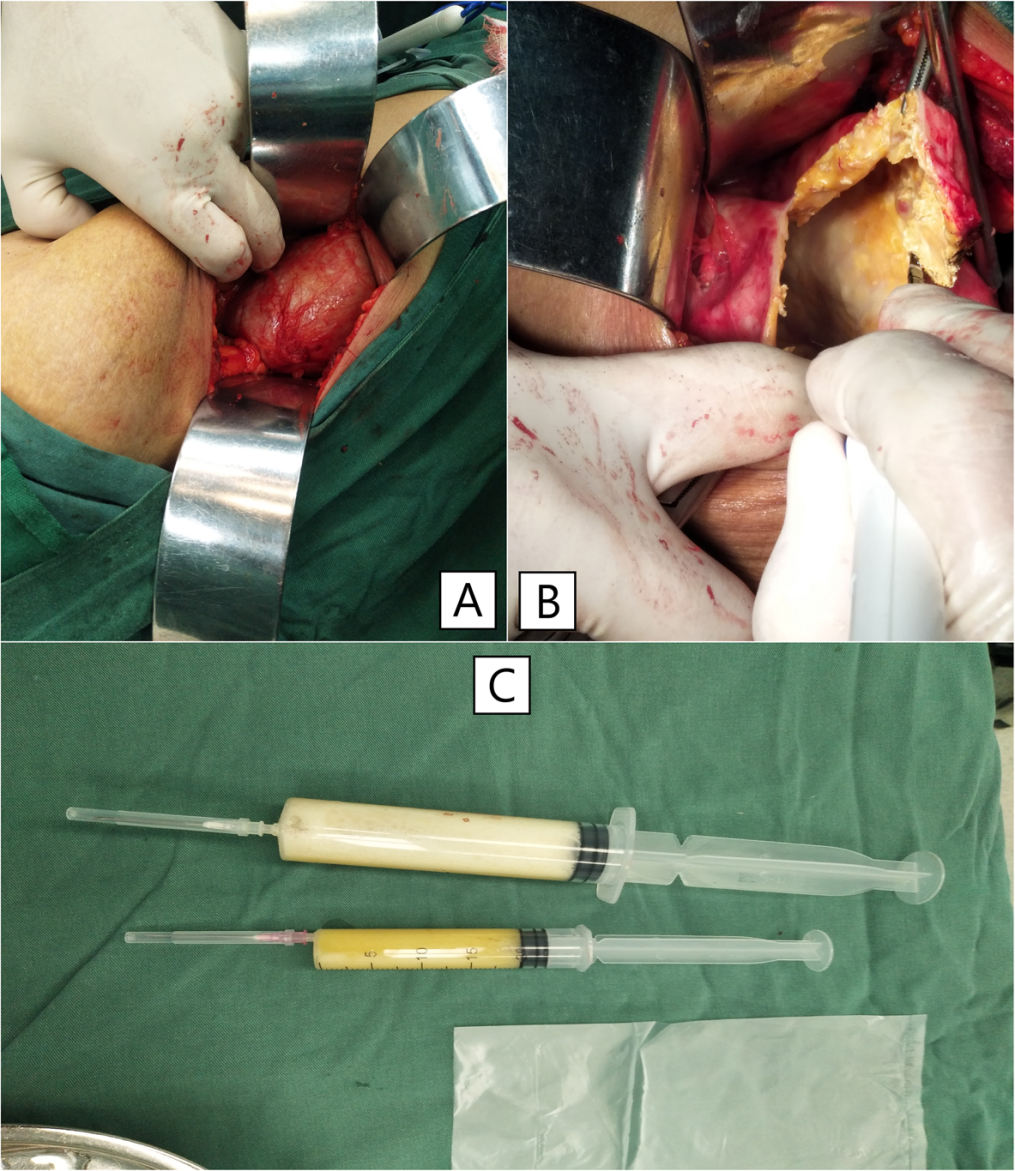
A chalky white cystic mass containing chalky white cystic fluid that was aspirated using two syringes

**Fig. 7:**
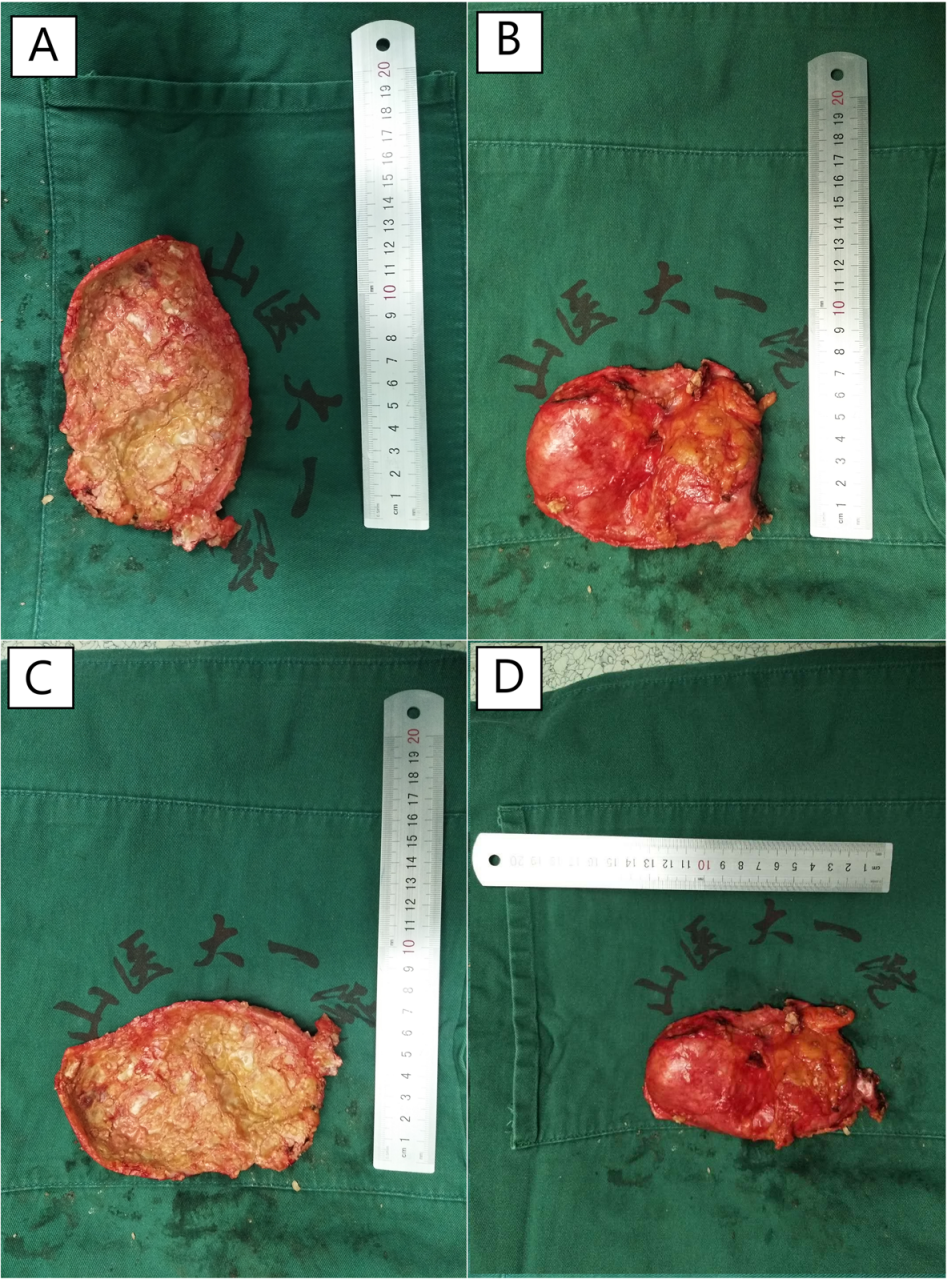
Chalky white cystic wall

**Fig. 8:**
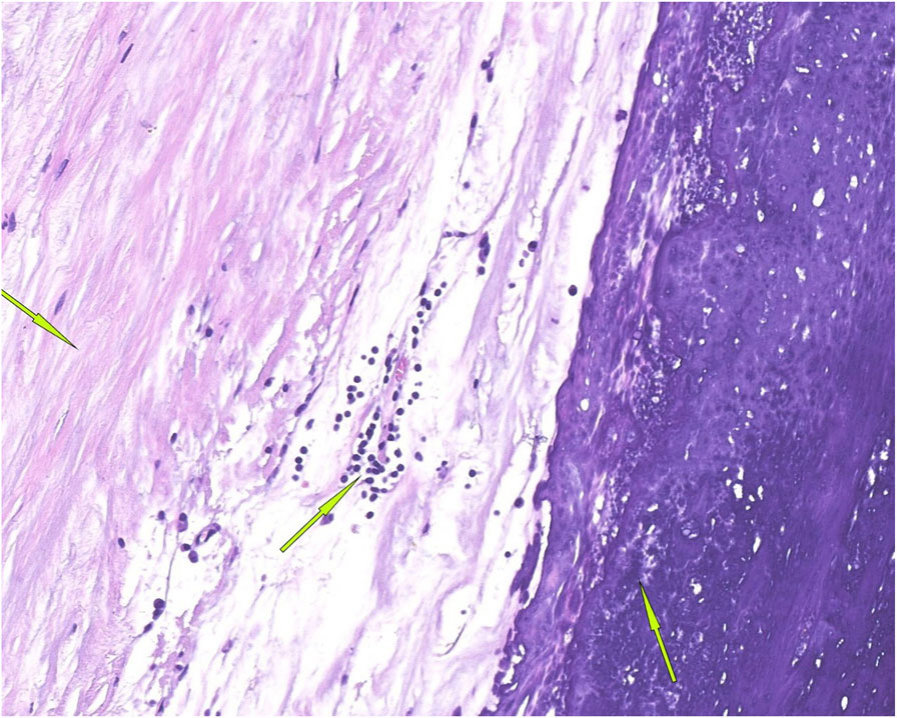
Representative images of cystic wall fibrosis, calcification and inflammatory cell infiltration (arrows: haematoxylin and eosin staining, magnification X 200)

**Fig. 9:**
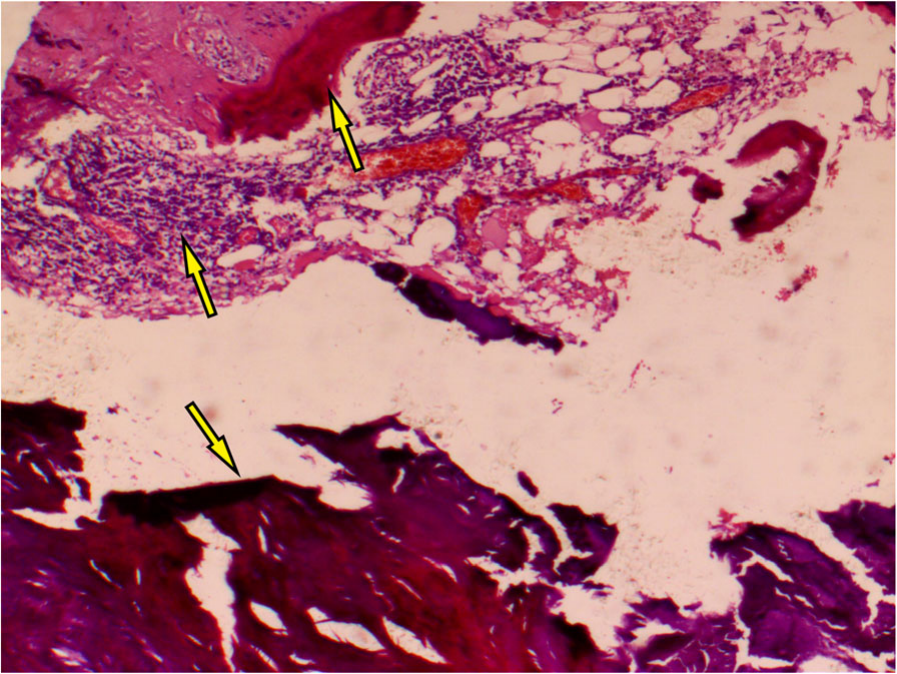
Representative images of biopsy samples showing the presence of ossification and bone marrow formation in the cyst wall (arrows; haematoxylin and eosin staining, magnification X 40)

To relieve the symptoms of soreness on the right side of the lumbar region, the patient underwent surgical resection of this lesion under general anaesthesia without partial removal of the renal parenchyma. During the operation, a hard and fluctuant cystic mass measuring 11.5 × 6.9 × 5.1 cm was found on the lateral side of the right kidney, which contained approximately 400 mL of chalky white fluid (Fig. 6). Before incising the renal mass, 70 mL cystic fluid was aspirated using the syringes (Fig. 6) and tested during the operation. No cancer cells or *Mycobacterium tuberculosis* were found in the smear test, which excluded the possibility of tumour and tuberculous abscess and indicated that the lesion was benign. Therefore, the cystic wall was opened by incision, and chalky calcifications were found in the inner layer of the cystic wall. The cystic wall was removed, and the cyst was not connected with the renal collecting system (Fig. 7). Postoperative culture of *M. tuberculosis*, aerobic bacteria and anaerobic bacteria in the cystic fluid was negative. The pathological observation showed the presence of fibrosis, calcification, infiltrated inflammatory cells, ossification and bone marrow formation in the cystic wall, so the patient was diagnosed with right renal cystic TC with ossification and bone marrow formation (Figs. 8 and 9). No recurrence was detected 1 year after surgery, and the patient’s quality of life was greatly improved.

## Discussion and conclusions

TC is a condition where calcium deposition tends to occur in soft tissues around the large joints, forming tumour-like nodular calcifications. Here, we describe a case of cystic TC with ossification and bone marrow formation that occurred in the kidney. The first symptom was intermittent right lumbar pain, without fever, weakness or weight loss.

TC can be divided into two categories with hyperphosphatemia and without hyperphosphatemia. TC with hyperphosphatemia mainly involves mutations of three genes, namely, GALNT3, FGF23, and KL [2, 5, 6]. Mutations of these three genes would lead to abnormal coding and expression of proteins, resulting in dysfunction of phosphate reabsorption in renal tubules, which would cause TC. TC without hyperphosphatemia mainly involves gene mutations of SMAD9 [7, 8]. SAMD9, an IFN-γ reactive protein, can interact with RGL2 to reduce the expression of EGR1, which is a protein directly related to ectopic calcification and inflammation [7]. Since our patient did not have hyperphosphatemia, this case may be related to a gene mutation of SMAD9, which still needs more research to discuss. In addition, we speculated that the pathophysiological changes might be related to the local inflammation caused by trauma, resulting in excessive synthesis of collagen fibres around the vessels and adipose tissues around the kidney, which could lead to calcium deposition.

Pathologically, TC can be divided into active and inactive phases [9–11], which can occur simultaneously. Amorphous granular calcifications, macrophages, osteoclast-like giant cells, and chronic inflammatory cells were seen at the active stage of TC, while calcifications were surrounded by dense fibrous tissues at the inactive stage. In this case, the pathological examination showed the presence of fibrous tissues, calcium deposition and infiltrated inflammatory cells in the calcified wall around the lesion, and especially the presence of ossification and bone marrow formation in the calcified wall. Chalky cystic fluid and granular calcification is surrounded by the circular calcified wall. These characteristics were distinctly different from the calcification of the simple renal cyst wall. After consultation with the relevant pathologists, this patient was diagnosed with renal cystic TC with ossification and bone marrow formation at the inactive stage. In addition, we only performed surgical resection of the lesion in this case and did not remove part of the renal parenchyma. During the 1-year follow-up, the prognosis for the patient was quite good, and no related complications or recurrence were found. We expect that this case report can provide valuable insights into the diagnosis and treatment of similar diseases to avoid unnecessary large-scale resection and reduce harm to patients. Some types of TC can recur after surgery [2, 10], even causing greater damage to the body; consequently, long-term follow-up is required for patients presenting renal cystic TC with ossification and bone marrow formation.

Renal cystic TC with ossification and bone marrow formation is a rare benign disease that needs to be differentiated from kidney stones, renal tuberculosis, renal cysts with calcified walls, and tumours. Complete surgical resection of the lesion is recommended, and there is no need to remove part of the renal parenchyma. The limitation of this paper is that certain laboratory tests and gene tests were not performed due to the initial lack of understanding of this new disease, and therefore, renal cystic TC still requires more research for further discussion.

## Data Availability

All data supporting our findings are contained within the manuscript.

## Abbreviations

TC: Tumoural calcinosis
CT: Computed tomography
KUB: Plain film of kidney ureters and bladder
IVU: Intravenous urography
